# Single-Crystalline High-Quality β‑Ga_2_O_3_ Pseudosubstrate on Sapphire through Sputtering for Epitaxial Deposition

**DOI:** 10.1021/acsaenm.6c00439

**Published:** 2026-06-02

**Authors:** Guangying Wang, Shuwen Xie, William Brand, Saleh Ahmed Khan, Ahmed Ibreljic, Darryl Shima, Yueying Ma, Brahmani Challa, Surjava Sanyal, Fikadu Alema, Andrei Osinsky, Anhar Bhuiyan, Ganesh Balakrishnan, Shubhra S. Pasayat

**Affiliations:** † Department of Electrical and Computer Engineering, 489565 University of Wisconsin-Madison, Madison, Wisconsin 53706, United States; ‡ 597379 Agnitron Technology Incorporated, Chanhassen, Minnesota 55317, United States; § Department of Electrical and Computer Engineering, 14710 University of Massachusetts Lowell, Lowell, Massachusetts 01854, United States; ∥ Center for High Technology Materials, 1104 University of New Mexico, Albuquerque, New Mexico 87106, United States

**Keywords:** sputtering, oxide, annealing, ultrawide bandgap, semiconductors, synthesis

## Abstract

Solid-phase epitaxy (SPE) of β-Ga_2_O_3_ thin films by radio frequency (RF) sputtering, followed by crystallization through high-temperature postdeposition annealing, is employed on sapphire substrates, yielding a high-quality pseudosubstrate for subsequent buffer growth via MOCVD and LPCVD. Low roughness (<0.5 nm) and sharp single-crystalline diffraction peaks corresponding to the (−201), (−402), and (−603) reflections of β-Ga_2_O_3_ were observed in the SPE β-Ga_2_O_3_ film and the subsequent epitaxial buffer layer. N-doped Ga_2_O_3_ film grown by LPCVD on SPE Ga_2_O_3_ film showed step-assisted growth mode with reasonable electronic behavior, with 45 cm^2^/V·s mobility at a bulk carrier concentration of 1.3 × 10^17^ cm^–3^. These results suggest that SPE Ga_2_O_3_ is a promising pathway to advance the development of β-Ga_2_O_3_ on foreign substrates.

## Graphical abstract

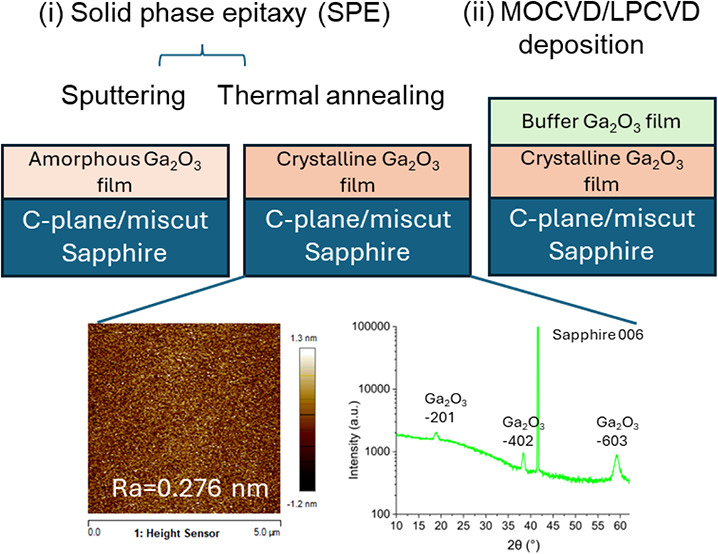

## Introduction

Gallium oxide (Ga_2_O_3_) has recently emerged as a highly promising semiconductor for next-generation power electronics. Its ultrawide bandgap (∼4.8 eV), high dielectric constant, and extremely high breakdown electric field (∼8 MV/cm) provide the theoretical basis for devices with superior power-handling capabilities compared to conventional wide-bandgap semiconductors such as silicon carbide (SiC) and gallium nitride (GaN).^1^ These attributes make Ga_2_O_3_ attractive for high-voltage transistors, Schottky diodes, and other high-power applications.

Despite these advantages, two fundamental challenges limit their practical deployment. First, the intrinsic thermal conductivity of Ga_2_O_3_ is relatively low (11–27 W·m^–1^·K^–1^, depending on the phase and orientation), which restricts heat dissipation and directly impacts device reliability, efficiency, and lifetime under high-current operation.^2^ Second, bulk Ga_2_O_3_ substrates, although commercially available, are expensive, with 4- and 6-in. diameter wafers still under development, posing obstacles for both research scalability and industrial adoption.^3–5^ These issues have motivated intensive exploration of thin-film growth on foreign substrates, combining the favorable properties of Ga_2_O_3_ with the thermal and mechanical advantages of scalable platforms.

Several epitaxial techniques have been investigated for this purpose, including Metal–Organic Chemical Vapor Deposition (MOCVD),^6–15^ Molecular Beam Epitaxy (MBE),^16–20^ and Radio-Frequency (RF) Magnetron Sputtering.^3,21–24^ MOCVD and MBE are capable of producing high-quality homoepitaxial films with well-controlled doping and layer structures. However, when adapted for heteroepitaxy, these methods generally require a nucleation stage to accommodate lattice mismatch, which complicates growth and often results in rough, defect-prone films, particularly at small thicknesses.^25^ In contrast, RF Magnetron Sputtering offers a simpler and more industrially scalable route for fabricating Ga_2_O_3_ layers. While it is less suited for building complex device heterostructures, sputtering can generate uniform and smooth films with large-area compatibility, making it highly attractive as a foundation for subsequent device fabrication.^3,21–24^ This technology has been widely utilized in other III–V group template development, such as sputtered AlN as a nucleation layer with GaN or AlN buffer layers to create templates on sapphire substrates.^26–29^

In this work, we propose a two-step pathway to address the material and integration challenges, as shown in Figure 1. First, Ga_2_O_3_ thin films are deposited on sapphire substrates by RF sputtering and subsequently crystallized through high-temperature postdeposition annealing, yielding epitaxial-quality films via solid-phase epitaxy (SPE). These sputtered and subsequently postannealed templates serve as high-quality seed layers with improved crystalline order and reduced defect density compared with Ga_2_O_3_ direct growth on sapphire with CVD. In the second step, thicker device-grade Ga_2_O_3_ layers are grown on these SPE templates using MOCVD and low-pressure chemical vapor deposition (LPCVD). MOCVD provides precise epitaxial control and is widely used for large-area production, while LPCVD enables higher growth rates that are suitable for thicker film deposition. These deposition techniques were employed to verify the epitaxial compatibility of SPE Ga_2_O_3_ pseudosubstrates, leveraging the improved surface morphology and crystalline quality of the underlying SPE templates. The successful epitaxial growth of Ga_2_O_3_ films on these templates demonstrates the feasibility of this pseudosubstrate approach for scalable device fabrication. This strategy provides a promising pathway toward scalable integration of Ga_2_O_3_ power electronic devices on foreign substrates.

**Figure 1:**
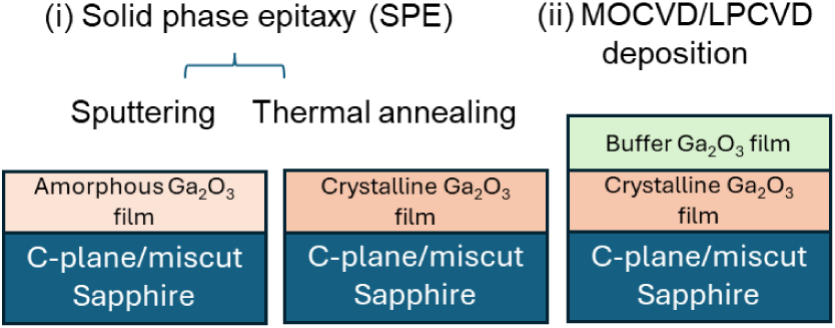
Schematic illustration of the two-step growth pathway: (i) formation of crystalline β-Ga_2_O_3_ on sapphire substrates by SPE, followed by (ii) deposition of the β-Ga_2_O_3_ buffer layer by MOCVD or LPCVD.

## Experiment

For the preparation of SPE β-Ga_2_O_3_ on sapphire substrates, an amorphous 25 nm Ga_2_O_3_ layer was first deposited on both c-plane sapphire and 6° miscut sapphire (toward the m-plane) using on-axis RF magnetron sputtering. The sputtering target was Ga_2_O_3_ with 99.99% purity, purchased from AEM deposition. The deposition was carried out at a substrate temperature of 200 °C under a chamber pressure of 15 mTorr, with 100 W RF power applied. The Ar flow rate was 20 sccm, with an O_2_ flow rate of 2 sccm, resulting in a sputter rate of around 25 nm/h. Crystallization of the sputtered Ga_2_O_3_ was then achieved by annealing in air at 850 °C in a muffle furnace. The resulting crystalline films were subsequently used as templates for buffer growth in MOCVD or LPCVD. For MOCVD-grown undoped β-Ga_2_O_3_ buffers on c-plane sapphire at 820 °C, triethylgallium (TEG) was employed as the Ga precursor at a molar flow rate of 9.2 μmol/min under an N_2_ carrier environment. The VI/III ratio was varied between 1000, 1500, and 3000, while maintaining a film thickness of approximately 200 nm. For the n-type LPCVD β-Ga_2_O_3_ growth, 6° off-miscut sapphire was utilized to suppress the island-nucleation-dominated growth mode expected on flat sapphire under a high growth rate (6–8 μm/hour), where the absence of atomic steps limits ordered adatom incorporation.^30^ The deposition was carried out at 1000 °C following the growth conditions described in ref. 31.

Atomic force microscopy (AFM) was carried out using a Bruker Dimension Icon system to examine the surface morphology of the β-Ga_2_O_3_ layers. The film thickness of the SPE β-Ga_2_O_3_ layers was determined by X-ray reflectivity (XRR), while crystal quality and phase identification were evaluated by high-resolution X-ray diffraction (XRD) ω–2θ scans using a Panalytical Empyrean diffractometer and scanning transmission electron microscopy (HRTEM/STEM) with an aberration-corrected Transmission Electron Microscope JEOL NEOARM 200CF. The surface morphology of the LPCVD-grown n-type β-Ga_2_O_3_ films on SPE sapphire substrates was further characterized by scanning electron microscopy (SEM). Electrical properties, including electron concentration and mobility, were measured on the same LPCVD-grown films using Hall effect measurements.

## Results

The AFM images of 25 nm SPE crystalline β-Ga_2_O_3_ grown on c-plane sapphire substrate and miscut sapphire substrate are shown in Figure 2. For a 1 × 1 μm^2^ scan, the surface roughness was 0.332 nm on the c-plane sapphire substrate and 0.406 nm on the miscut sapphire substrate. At a larger 5 × 5 μm^2^ scan size, the corresponding values were 0.276 and 0.356 nm, respectively. Under identical sputtering and postannealing conditions, the crystalline β-Ga_2_O_3_ film on the planar sapphire substrate consistently exhibited lower roughness than the film on the miscut sapphire substrate. This difference is attributed to the higher density of atomic steps on the miscut substrate, which propagate into the epitaxial film and enhance step-related height variations. In both cases, the RMS roughness remained below 0.5 nm, confirming atomically smooth surfaces comparable to those of bulk β-Ga_2_O_3_. Moreover, XRD patterns of crystalline β-Ga_2_O_3_ films grown on both c-plane sapphire substrate and miscut sapphire substrate exhibited only the (−201), (−402), and (−603) reflections of β-Ga_2_O_3_ together with the (006) reflection of sapphire. Annealing was performed at 850 °C, which stabilized the β-Ga_2_O_3_ phase. To further verify crystallinity and epitaxial alignment, an off-axis φ-scan of the β-Ga_2_O_3_ (−401) reflection was performed, as shown in Figure 3. The φ-scan revealed 6-fold symmetric peaks, arising from rotational domains induced by the hexagonal symmetry of the sapphire substrate, thereby confirming the epitaxial nature and single-phase β-Ga_2_O_3_ growth.^32^

**Figure 2:**
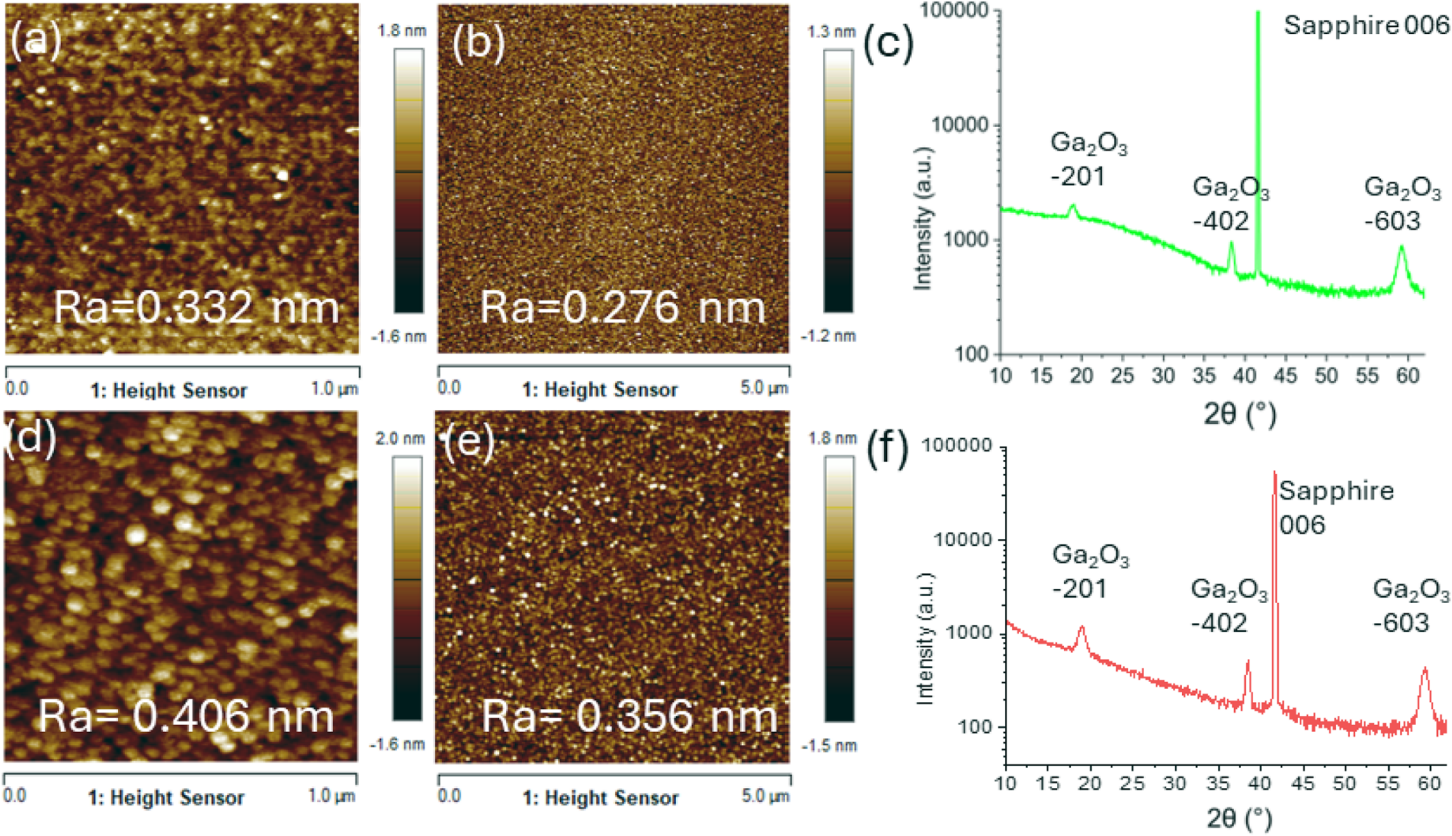
AFM scan of crystalline Ga_2_O_3_ on (a) planar sapphire substrate with a 1 × 1 μm^2^ scan size, (b) planar sapphire substrate with a 5 × 5 μm^2^ scan size, (d) 6° miscut sapphire substrate with a 1 × 1 μm^2^ scan size, and (e) 6° miscut sapphire substrate with a 5 × 5 μm^2^ scan size. XRD diffraction pattern of SPE Ga_2_O_3_ on (c) c-plane sapphire, and (f) miscut sapphire substrate.

**Figure 3:**
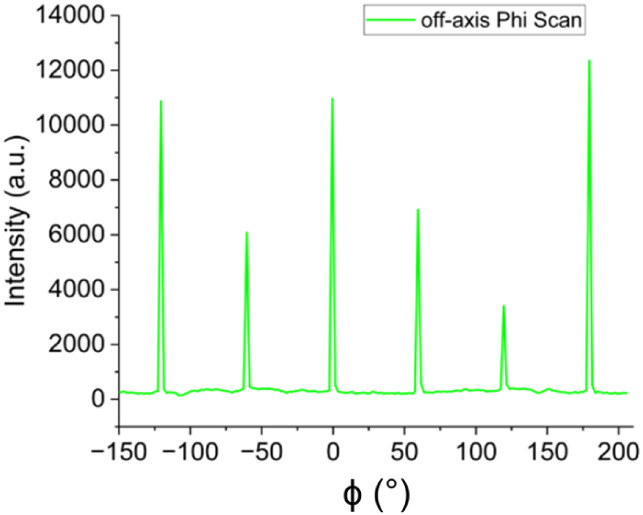
XRD off-axis φ-scan of SPE Ga_2_O_3_ on sapphire at β-Ga_2_O_3_ (−401).

The SPE-grown β-Ga_2_O_3_ layers were subsequently used as substrates for buffer growth. MOCVD was carried out on c-plane sapphire with the SPE β-Ga_2_O_3_ layer, while LPCVD was performed on miscut sapphire to take advantage of the faster growth rate achievable on this orientation. For both deposition methods, a bare sapphire substrate without the SPE layer was included as a reference for a fair comparison.

Figure 4(a–c) presents the AFM results of ∼250 nm-thick β-Ga_2_O_3_ buffer layers grown by MOCVD on SPE β-Ga_2_O_3_ on c-plane sapphire substrates under different VI/III ratios of 1000, 1500, and 3000. Results are summarized in Table 1. At a scan area of 5 × 5 μm^2^, the β-Ga_2_O_3_ grown directly on bare sapphire substrates exhibited surface roughness values of 3.87, 6.92, and 7.84 nm for VI/III ratios of 1000, 1500, and 3000, respectively. In contrast, when grown on SPE β-Ga_2_O_3_ on c-plane sapphire substrates, the roughness was consistently reduced to 3.74, 4.73, and 5.50 nm for the same VI/III ratios. These results indicate that buffer layers grown on SPE templates suppress facet development, particularly under lower VI/III ratios. VI/III ratios of 1000 showed the lowest surface roughness among all three VI/III conditions, possibly due to reduced gas-phase reaction.^33,34^ Across all three conditions, MOCVD-grown β-Ga_2_O_3_ buffers on SPE sapphire substrates consistently demonstrated lower surface roughness compared to those on bare sapphire substrates. The SPE template produced a larger absolute reduction in RMS roughness for the VI/III = 3000 sample, decreasing from 7.85 to 5.5 nm. In contrast, the VI/III = 1000 sample already had a smoother baseline surface, with an RMS roughness of 3.87 nm, so the additional reduction after introducing the SPE template, to 3.74 nm, was less pronounced.

**Figure 4:**
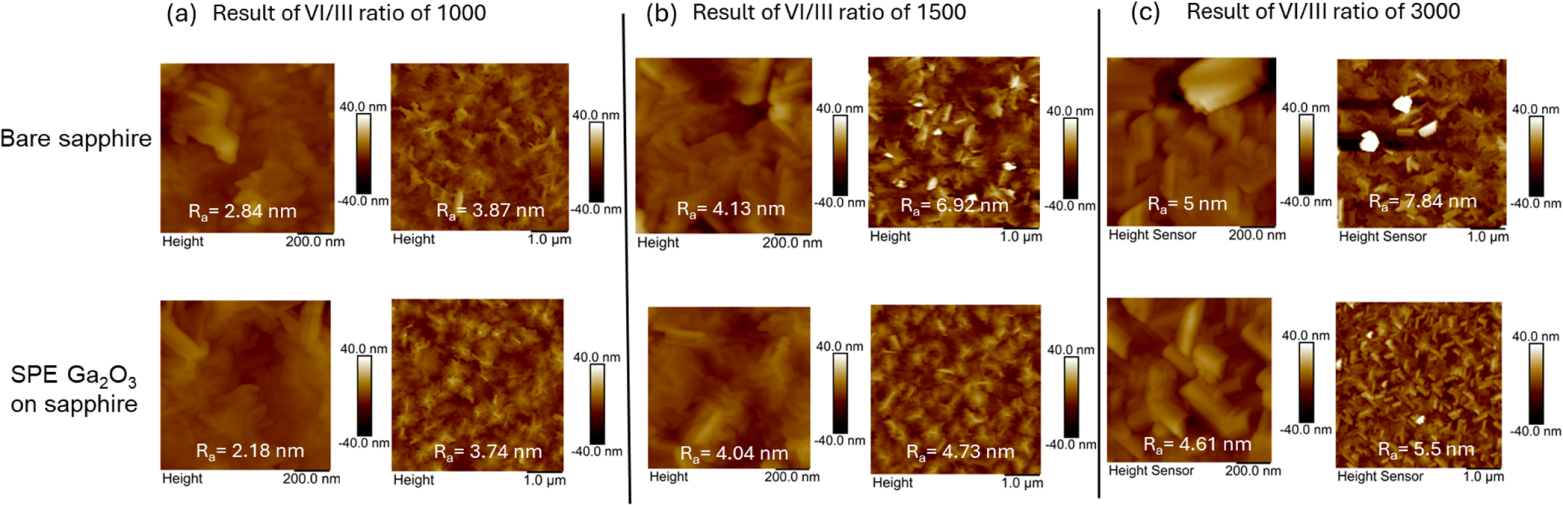
AFM results of MOCVD-grown Ga_2_O_3_ with VI/III ratios of (a) 1000, (b) 1500, and (c) 3000.

**Table 1:** Summary of Ga_2_O_3_ Film Deposition Method, Substrates, Growth Conditions, and RMS Roughness of 1 × 1 μm^2^ and 5 × 5 μm^2^

Growth method	Substrate/template	VI/III ratio	RMS roughness, 1 × 1 μm^2^ (nm)	RMS roughness, 5 × 5 μm^2^ (nm)
MOCVD Ga_2_O_3_	Bare c-plane sapphire	1000	2.84	3.87
MOCVD Ga_2_O_3_	SPE Ga_2_O_3_/c-plane sapphire	1000	2.18	3.74
MOCVD Ga_2_O_3_	Bare c-plane sapphire	1500	4.13	6.92
MOCVD Ga_2_O_3_	SPE Ga_2_O_3_/c-plane sapphire	1500	4.04	4.73
MOCVD Ga_2_O_3_	Bare c-plane sapphire	3000	5	7.84
MOCVD Ga_2_O_3_	SPE Ga_2_O_3_/c-plane sapphire	3000	4.61	5.5

The structural quality was further confirmed by XRD analysis, as shown in Figure 5. Films grown on SPE β-Ga_2_O_3_ on a c-plane sapphire substrate exhibited sharp diffraction peaks corresponding only to the (−201), (−402), (−603), and (−804) planes of β-Ga_2_O_3_, together with the (006) reflection of sapphire. In contrast, MOCVD-grown β-Ga_2_O_3_ on bare sapphire showed additional parasitic peaks, indicative of secondary phases or misoriented grains.

**Figure 5:**
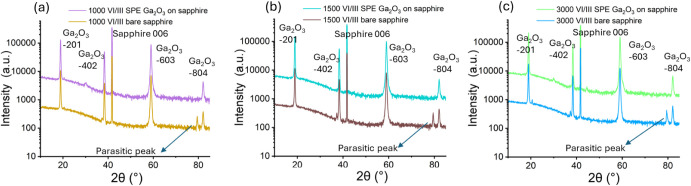
XRD results of MOCVD-grown Ga_2_O_3_ with VI/III ratios (with an offset in intensity to show the parasitic peak) of (a) 1000, (b) 1500, and (c) 3000 on SPE Ga_2_O_3_ on sapphire substrate and bare sapphire substrate.

Furthermore, a TEM study was performed on Ga_2_O_3_ on an SPE Ga_2_O_3_ on sapphire sample, as shown in Figure 6. Clear lattice orientation can be observed both at the SPE Ga_2_O_3_ to sapphire interface and MOCVD Ga_2_O_3_ to SPE Ga_2_O_3_ interface (identified from a lower-magnification scan), indicating the preferred −201 orientation of the Ga_2_O_3_ film. However, defects are also observed in Figure 6(b) in the SPE Ga_2_O_3_ film, indicating that further optimization is needed to improve uniformity. These findings in XRD and TEM demonstrate that the SPE β-Ga_2_O_3_ template provides a clear advantage in both crystalline quality and surface morphology compared to direct growth on bare sapphire substrates.

**Figure 6:**
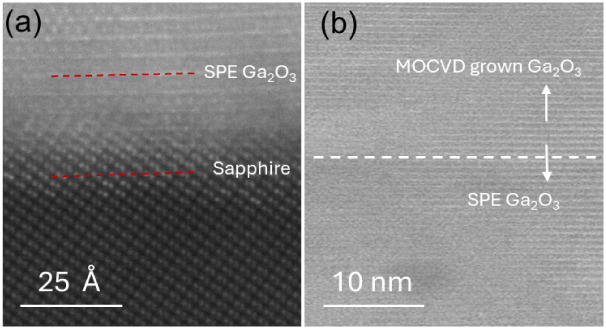
TEM results of MOCVD-grown Ga_2_O_3_ on SPE Ga_2_O_3_ on sapphire with a VI/III ratio of 1000 at the (a) SPE Ga_2_O_3_ and sapphire interface, with a red dashed line showing lattice orientation matching, and (b) MOCVD Ga_2_O_3_ on SPE Ga_2_O_3_ on sapphire, with a white dashed line showing the Ga_2_O_3_ interface.

The Field Emission Scanning Electron Microscopy (FESEM)-based characterization of the surface morphology and electrical transport characteristics of the LPCVD β-Ga_2_O_3_ films using Hall measurements, deposited on SPE Ga_2_O_3_ on 6° off-axis sapphire, is shown in Figure 7 (a). The film exhibited elongated surface features roughly aligned with the off-cut direction, which is characteristic of a step-assisted deposition mode. Similar features have been reported for LPCVD β-Ga_2_O_3_ on vicinal sapphire, where surface steps were suggested to reduce random island nucleation, suppress rotational variants, and promote lateral ordering.^30,31^ The SPE pseudosubstrate sample showed a similar morphology on the vicinal surface and yielded a mobility of 45 cm^2^/V·s at a bulk carrier concentration of 1.3 × 10^17^ cm^–3^, which is consistent with previously reported transport values for LPCVD Ga_2_O_3_ grown on 6° off-axis sapphire. The XRD results, shown in Figure 7 (b), further support these observations. The LPCVD-grown β-Ga_2_O_3_ film on bare miscut sapphire displayed parasitic peaks, whereas the film on the SPE template exhibited dominant β-Ga_2_O_3_ (−201), (−402), (−603), and (−804) reflections along with the sapphire (006) peak. For the film on the SPE template, additional weak polycrystalline peaks were detected, consistent with the SEM morphology that revealed faceting and mixed-orientation regions. However, these peaks were of very low intensity compared to the dominant (−201) reflection, indicating that they might originate from a thin fraction near the film surface. The AFM image shows a faceted surface morphology. The presence of these facets suggests nonuniform surface evolution during growth, likely associated with anisotropic growth rates along different crystallographic directions. Further LPCVD optimization is planned to suppress these features and achieve uniform single-phase films in order to further improve the film morphology and carrier mobility.

**Figure 7:**
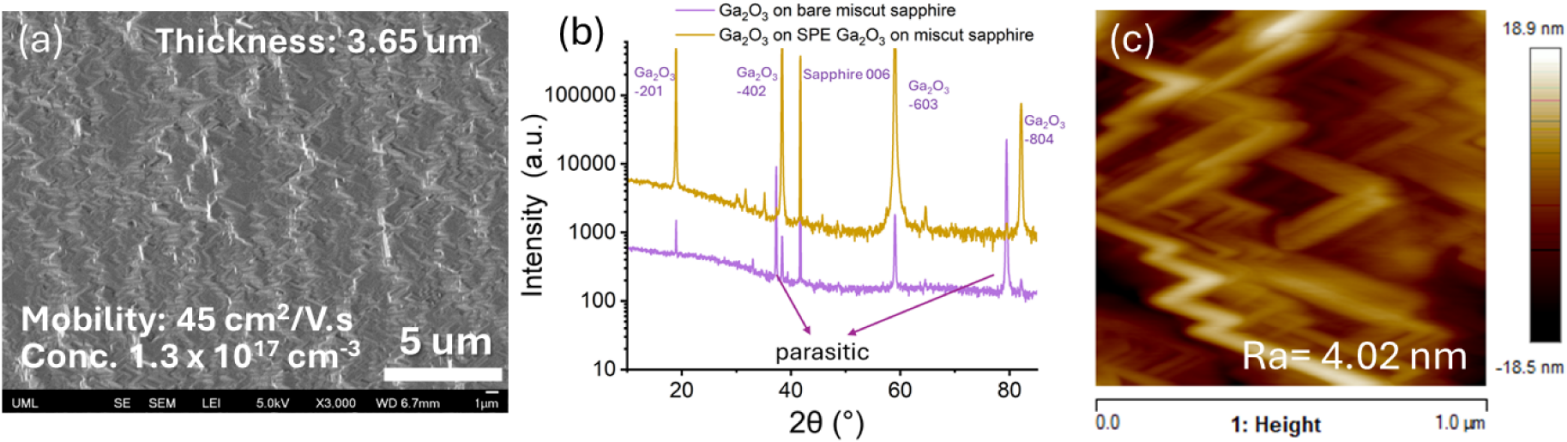
(a) Surface morphology and transport properties of LPCVD β-Ga_2_O_3_ grown films on SPE β-Ga_2_O_3_ template on 6° off-miscut sapphire, and (b) XRD results of LPCVD-grown Ga_2_O_3_ on SPE Ga_2_O_3_ on miscut sapphire substrate and on bare miscut sapphire substrate. (c) AFM results of LPCVD-grown Ga_2_O_3_ on SPE Ga_2_O_3_ on miscut sapphire substrate.

To evaluate the phase and in-plane epitaxial alignment, off-axis ϕ-scans of the β-Ga_2_O_3_ (−401) reflection were performed on four samples: MOCVD-grown Ga_2_O_3_ on bare c-plane sapphire, MOCVD-grown Ga_2_O_3_ on SPE-Ga_2_O_3_/sapphire, LPCVD-grown Ga_2_O_3_ on bare sapphire, and LPCVD-grown Ga_2_O_3_ on SPE-Ga_2_O_3_ sapphire, as shown in Figure 8. The two MOCVD-grown films show a 6-fold ϕ-scan pattern, which indicates multiple in-plane rotational variants related to the hexagonal symmetry of the c-plane sapphire substrate. By comparison, the LPCVD-grown films on 6° off-cut sapphire show fewer dominant ϕ peaks, suggesting stronger preferential in-plane orientation and partial suppression of rotational variants. This is consistent with previous reports that off-cut sapphire can reduce rotational-domain formation in heteroepitaxial β-Ga_2_O_3_ by lowering the effective in-plane symmetry of the growth surface and favoring selected epitaxial variants.^30,35,36^ The SPE nucleation layer does not appear to cause a clear additional reduction in the in-plane rotational-domain distribution beyond the effect of the off-cut substrate. This may be because the SPE layer improves phase selectivity and surface morphology, but its thickness is not sufficient to fully impose a single in-plane epitaxial registry during later MOCVD or LPCVD overgrowth.

**Figure 8:**
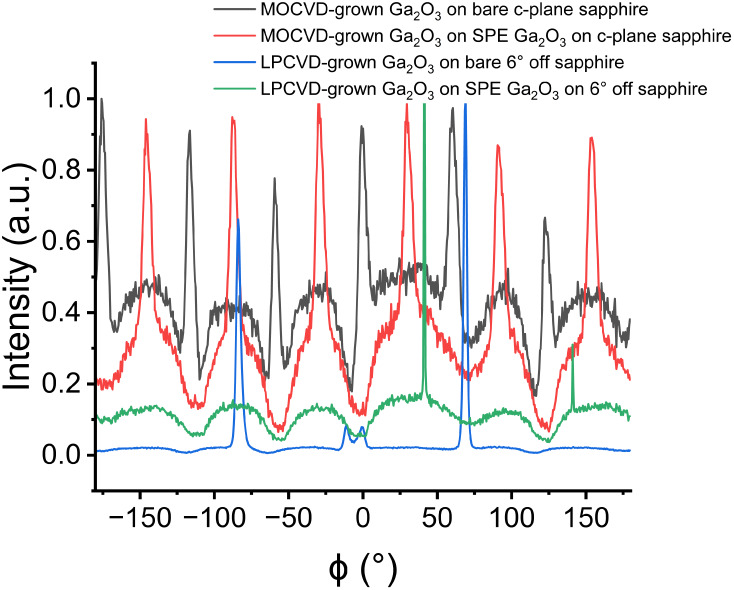
XRD off-axis φ-scan of MOCVD-grown Ga_2_O_3_ on bare sapphire, MOCVD-grown Ga_2_O_3_ on SPE Ga_2_O_3_ on sapphire, LPCVD-grown Ga_2_O_3_ on bare 6° off sapphire, and LPCVD-grown Ga_2_O_3_ on SPE Ga_2_O_3_ on 6° off sapphire at β-Ga_2_O_3_ (−401).

## Conclusion

In this work, we demonstrated the deposition of single-crystalline SPE β-Ga_2_O_3_ films on both c-plane and miscut sapphire substrates. The SPE β-Ga_2_O_3_ films exhibited subnanometer roughness (<0.5 nm) and sharp single-crystalline diffraction peaks corresponding to the (−201), (−402), and (−603) reflections of β-Ga_2_O_3_. Furthermore, both MOCVD-grown and LPCVD-grown β-Ga_2_O_3_ films deposited on SPE β-Ga_2_O_3_ on sapphire showed a clear reduction in parasitic peaks compared with reference films grown on coloaded sapphire substrates. LPCVD-grown n-type β-Ga_2_O_3_ films on SPE β-Ga_2_O_3_ on sapphire exhibited a step-assisted deposition mode with reasonable electron mobility and electron concentration.

By integrating physical vapor deposition and chemical vapor deposition, our approach combines the advantages of high scalability and smooth surface achieved through sputtering with the precise compositional control and superior electrical quality offered by CVD. As a result, we realize single-crystalline β-Ga_2_O_3_ films with excellent surface smoothness, strong structural integrity, and enhanced electrical performance. This combined strategy provides a promising pathway to advance the development of β-Ga_2_O_3_ for both fundamental research and practical device applications.
